# Supplementary material for: Long-Term Impact of D2 Lymphadenectomy during Gastrectomy for Cancer: Individual Patient Data Meta-Analysis and Restricted Mean Survival Time Estimation
Source: Cancers (Basel). 2024 Jan 19;16(2):424. doi: 10.3390/cancers16020424 (PMC10814228; doi:10.3390/cancers16020424)
Supplement: Supplementary file 1 [file cancers-16-00424-s001.zip › File S1.pdf]

Appendix 1

CENTRAL search strategy

#1 MeSH descriptor: [Stomach Neoplasms] explode all trees

#2 ((gastric or stomach) near/4 (cancer* or carcin* or malig* or tumor* or tumour* or neoplas* or adenocarcinoma)):ti,ab,kw (Word variations have been searched)

#3 #1 or #2

#4 MeSH descriptor: [Survival] explode all trees

#5 MeSH descriptor: [Survival Analysis] explode all trees

#6 (surviv* or DFS or DSS):ti,ab,kw (Word variations have been searched)

#7 MeSH descriptor: [Mortality] explode all trees

#8 MeSH descriptor: [Death] explode all trees

#9 (death* or died or mortality or mortalities or deceased or dead* or fatal* or lethal):ti,ab,kw (Word variations have been searched)

#10 #4 or #5 or #6 or #7 or #8 or #9

#11 #3 and #10

#12 MeSH descriptor: [Lymph Nodes] explode all trees

#13 MeSH descriptor: [Lymph Node Excision] explode all trees

#14 (lymphadenectom* or lymph node* or LN or LND or LNE):ti,ab,kw (Word variations have been searched)

#15 #12 or #13 or #14

#16 #11 and #15

MEDLINE search strategy

1 exp Stomach Neoplasms/

2 ((gastric or stomach) adj4 (cancer* or carcin* or malig* or tumor* or tumour* or neoplas* or adenocarcinoma)).ti,ab,kw.

3 1 or 2

4 exp Disease-Free Survival/ or exp Survival Analysis/ or exp Survival/ or exp Survival Rate/

5 (surviv* or DFS or DSS).ti,ab,kw.

6 exp Hospital Mortality/ or exp Mortality/

7 exp Death/

8 (death* or died or mortality or mortalities or deceased or dead* or fatal* or lethal*).ti,ab,kw.

9 or/4-8

10 3 and 9

11 exp Lymph Node Excision/ or exp Lymph Nodes/

12 (lymphadenectom* or lymph node* or LN or LND or LNE).ti,ab,kw.

13 11 or 12

14 10 and 13

15 randomized controlled trial.pt.

16 controlled clinical trial.pt.

17 random*.mp.

18 trial.ab.

19 groups.ab.

20 or/15-19

21 14 and 20

22 case report/

23 case study/

24 exp animals/ not humans/

25 or/22-24

26 21 not 25

EMBASE search strategy

1 exp stomach tumor/

2 ((gastric or stomach) adj4 (cancer* or carcin* or malig* or tumor* or tumour* or neoplas* or adenocarcinoma)).ti,ab,kw.

3 1 or 2

4 exp disease free survival/ or exp survival rate/ or exp cancer survival/ or exp disease specific survival/ or exp event free survival/ or exp cancer specific survival/ or exp survival/ or exp failure free survival/ or exp overall survival/

5 (surviv* or DFS or DSS).ti,ab,kw.

6 exp mortality/ or exp cancer mortality/

7 exp death/

8 (death* or died or mortality or mortalities deceased or dead* or fatal* or lethal*).ti,ab,kw.

9 or/4-8

10 3 and 9

11 exp lymph node/

12 exp lymph node dissection/

13 (lymphadenectom* or lymph node* or LN or LND or LNE).ti,ab,kw.

14 11 or 12 or 13

15 10 and 14

16 random$.mp.

17 double-blind$.mp. or blind$.tw.

18 clinical trial:.mp.

19 16 or 17 or 18

20 15 and 19

21 Case study/

22 case report/

23 exp animal/ not human/

24 21 or 22 or 23

25 20 not 24
