# Supplementary material for: Long-Term Impact of D2 Lymphadenectomy during Gastrectomy for Cancer: Individual Patient Data Meta-Analysis and Restricted Mean Survival Time Estimation
Source: Cancers (Basel). 2024 Jan 19;16(2):424. doi: 10.3390/cancers16020424 (PMC10814228; doi:10.3390/cancers16020424)
Supplement: Supplementary file 1 [file cancers-16-00424-s001.zip › Suppl Table 1.pdf]

Author, year, contry	RCT design	Method of randomization	Surgeons' eligibility	Surgical quality control	Blinding	Power analysis	Participating surgeons training	Inclusion criteria	Exclusion criteria	SG vs. TG
Degiuli et al. [34]	superiority	block randomization (fixed block size n=10); intraoperative randomization	Multicentric; experienced trained surgeons*	monitorization of number and site of LN + regular meetings	P: not blind. S: not blind. Outcome was assessed blind of treatment allocation	Yes, AP	* Surgeon from the reference centre (MD) stayed at the National Cancer Center Hospital, Tokyo, to learn the D2 dissection from a specialist Japanese surgeon (MS). He was given didactic videos, papers and explanatory booklets edited by Japanese authors. MD became the supervisor of the trial. The IGCSG was set up in April 1994 and nine institutions participated. Each centre had two surgeons attending all the operations. Before starting the trial, several meetings were organised among participating centres to explain the terminology, to debate the proper indications and demonstrate the surgical technique. At least one of the two surgeons of each participating institution observed the first 10 procedures in this trial, which were performed at the reference centre. Afterwards, MD attended the first three operations performed at each institution.	histologically proven gastric cancer preoperatively staged as potentially resectable with curative intent, and did not carry any severe comorbidities involving cardiorespiratory, renal or metabolic system which could preclude safe D2 resection	older than 80, other cancer, previous gastrectomy, emergency surgery	*a distal gastrectomy was done when the proximal edge of the tumour was more than 3 cm from the cardias in early gastric cancer (EGC) and in Bormann type 1 and 2 locally advanced gastric cancer (AGC). Otherwise, and in case the tumour was located close to the greater curvature, beyond Demel's point, as well as in patients with linitis plastica, a total gastrectomy was recommended.

Songun et al., [33]	nr	block randomization (fixed block size n=6); intraoperative randomization	Multicentric; experienced trained surgeons*	monitorization of number and site of LN + regular meetings	P: ?. S: not blind. Outcome was not assessed blind to group allocation.	Yes, AP	* Participating surgeons received a videotape and an instruction booklet about the technique and were instructed in the operating theatre by an expert gastric cancer surgeon. The expert was present during the first 4 months of intake, which served as an instruction period, and regularly thereafter. All surgeries involving D2 dissection were attended by one of 11 surgeons who had been trained in D2 dissection. The study coordinator attended nearly all D1 procedures. The supervising surgeons monitored the technique and the extent of lymph-node dissection. After the surgery, the perigastric tissue was divided into lymph-node stations and fresh specimens were sent to the pathologist. All locations dissected not en-bloc were prepared and labelled by the surgeon.	patients with histologically proven adenocarcinoma of the stomach without evidence of distance metastasis, and were in adequate physical condition for D1 or D2 lymphadenectomy	older than 85, other cancer, previous gastrectomy	*proximal margin of 5cm for distal gastrectomy
Wu et al., [32]	nr	permuted block randomization (n=4, 6 or 8 - randomly chosen by computer algorithm)	Single center; experienced trained surgeons (min 25 D2 resections)*	monitorization of resected LN + regular meetings	No	Yes, AP	A participating surgeon (CWW) learned the operative technique from several diff erent medical centres in Japan, and set-up our standard procedure. Two other surgeons (SSL and MCH) gained experience of this standard procedure by serving as assistants and operators under the training of CWW. Before the trial, all participating surgeons had done at least 25 independent D3 resections.	patients with histologically proven, potentially curable gastric adenocarcinoma, and had physical fitness suitable for elective operation of either type of lymphadenectomy.	older than 75, other cancer, previous gastrectomy, previous RCT, esophageal involvement, early gastric cancer, nodal involvement - paraaortic or hepatoduodenal, distant metastasis	*proximal margin of 3cm (well-defined) and 5cm (diffuse) for distal gastrectomy

Cuschieri et al.,[31]	nr	random permutated block randomization; intraoperative randomization	Multiple surgeons (32)	trial monitoring committee	-	Yes, AP	Operative booklet and video tapes were produced	patients with histologically proven, and potentially curable, gastric carcinoma	younger than 20, previous gastric surgery, coexisting cancer, comorbiditie s	*proximal margin of 2.5cm for distal gastrectomy
Robertson et al., [30]	nr	random numbers generated on a computer; intraoperative randomization	-	-	-	UP		gastric antrum tumor, no peritoneal or liver metastases, that allows a safe margin for a radical subtotal gastrectomy	older than 75, serios comorbiditie s	

Supplementary Table 1. Randomized Clinical Trials (RCTs) quality evaluation. P patient, S surgeon, Y yes, UP under-powered. AP appropriately powered. SG subtotal gastrectomy.TG total gastrectomy.
