# Supplementary material for: Long-Term Impact of D2 Lymphadenectomy during Gastrectomy for Cancer: Individual Patient Data Meta-Analysis and Restricted Mean Survival Time Estimation
Source: Cancers (Basel). 2024 Jan 19;16(2):424. doi: 10.3390/cancers16020424 (PMC10814228; doi:10.3390/cancers16020424)
Supplement: Supplementary file 1 [file cancers-16-00424-s001.zip › Suppl Table 2.pdf]

Table S2. The GRADE certainty of evidence for OS, CSS, and DFS.

Author(s): Aiolfi A, et al
Question:
Setting:
Bibliography:

Certainty assessment							№ of patients		Effect		Certainty
№ of studies	Study design	Risk of bias	Inconsistency	Indirectness	Imprecision	Other considerations	[intervento]	[confronto]	Relative (95% CI)	Absolute (95% CI)	
5	randomised trials	not serious	not serious	not serious	not serious	all plausible residual confounding would reduce the demonstrated effect			-	SMD **1.8 SD higher** (4.2 lower to 0.7 higher)	⨁⨁⨁⨁ High
3	randomised trials	not serious	not serious	not serious	not serious	all plausible residual confounding would reduce the demonstrated effect			-	SMD **1.2 SD higher** (3.9 lower to 5.7 higher)	⨁⨁⨁⨁ High
3	randomised trials	serious	not serious	not serious	not serious	publication bias strongly suspected all plausible residual confounding would reduce the demonstrated effect			-	SMD **0.8 SD higher** (1.7 lower to 3.4 higher)	⨁⨁⨁◯ Moderate

CI: confidence interval; **SMD:** standardised mean difference
