# Supplementary figures and images for: Long-Term Impact of D2 Lymphadenectomy during Gastrectomy for Cancer: Individual Patient Data Meta-Analysis and Restricted Mean Survival Time Estimation
Source: Cancers (Basel). 2024 Jan 19;16(2):424. doi: 10.3390/cancers16020424 (PMC10814228; doi:10.3390/cancers16020424)

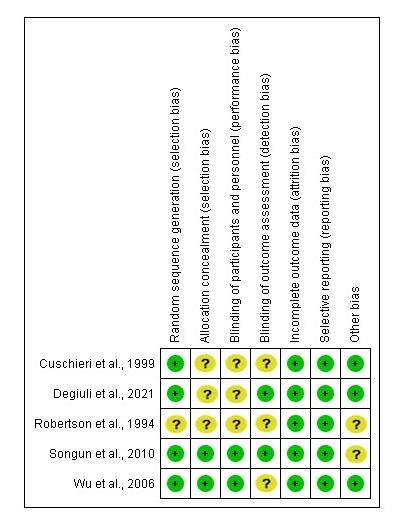

Supplement: Supplementary file 1 [file cancers-16-00424-s001.zip › Suppl Figure 1 - risk of bias def.tiff]

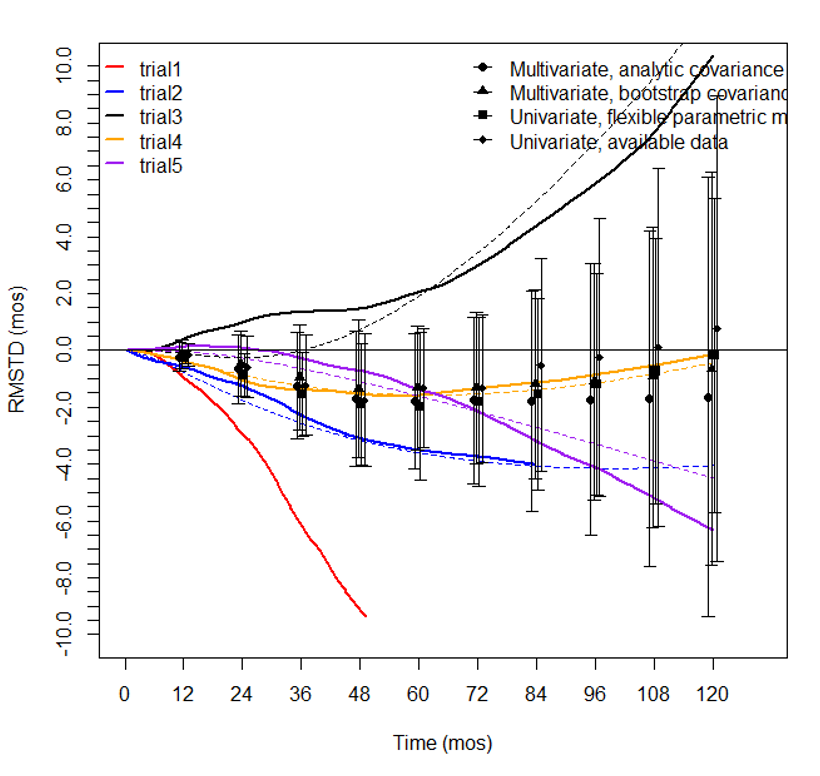

Supplement: Supplementary file 1 [file cancers-16-00424-s001.zip › Suppl Figure 2 def - OS.tiff]

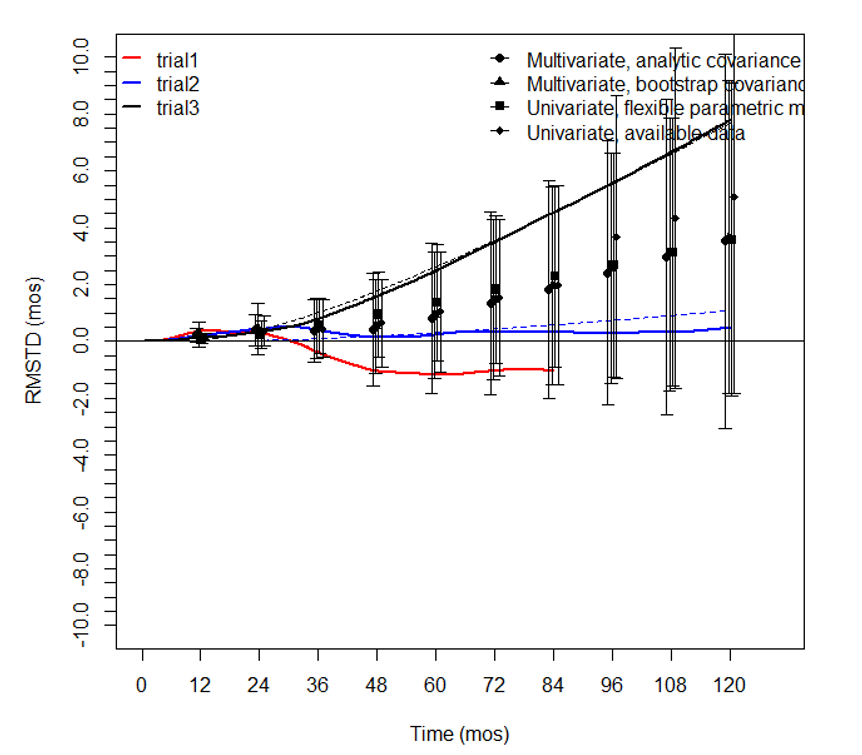

Supplement: Supplementary file 1 [file cancers-16-00424-s001.zip › Suppl Figure 3 def - CSS.tiff]

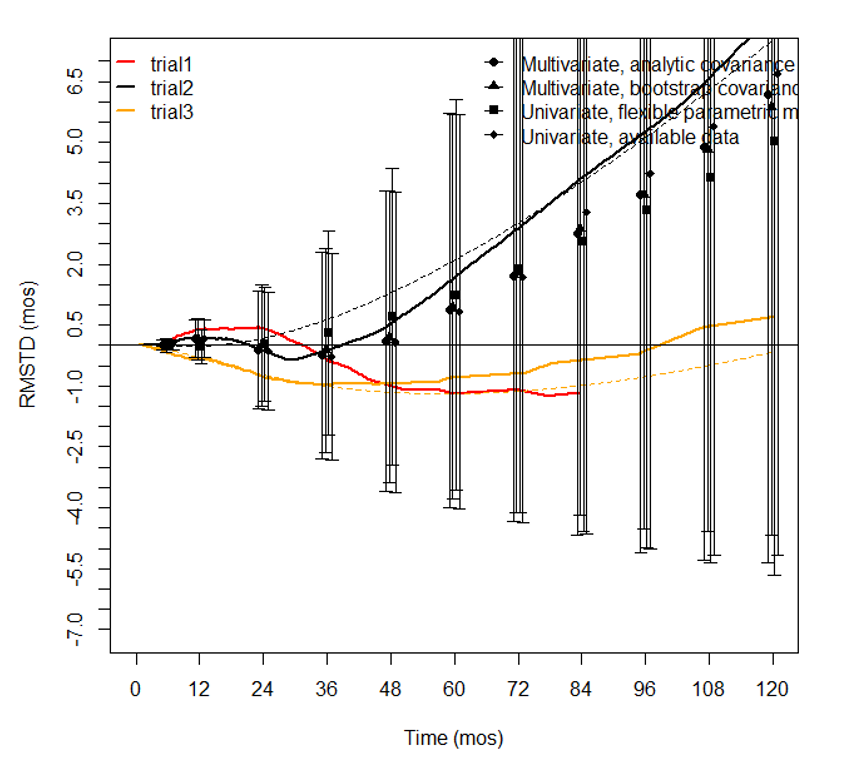

Supplement: Supplementary file 1 [file cancers-16-00424-s001.zip › Suppl Figure 4 def - DFS.tiff]
